# Saccadic Eye Movements Attenuate Postural Sway but Less in Sleep-Deprived Young Adults

**DOI:** 10.3389/fspor.2020.00097

**Published:** 2020-08-12

**Authors:** Ivan E. Pinto Vargas, Lucas E. Bicalho, Sérgio T. Rodrigues, José A. Barela

**Affiliations:** ^1^Faculty of Psychomotor, Health, Education and Sports, Salesiana University of Bolivia, La Paz, Bolivia; ^2^School of Physical Education, Physiotherapy and Occupational Therapy, Federal University of Minas Gerais, Belo Horizonte, Brazil; ^3^Faculty of Science, São Paulo State University, Bauru, Brazil; ^4^Institute of Biosciences, São Paulo State University, Rio Claro, Brazil

**Keywords:** postural balance, saccades, sleepiness, visual fixation, adults

## Abstract

Sleep deprivation affects the performance of postural control and several other aspects related to attentional mechanisms that may alter sensory cue acquisition strategies. This study aimed to examine the possible effects of horizontal saccades and ocular fixation on a target in the performance of postural control in young adults with sleep deprivation. Twenty-six adults formed two groups, tested in two evaluations. In the first evaluation, participants slept normally on the night before. In the second evaluation, 13 participants were sleep deprived (SD) and 13 slept normally (control group [CG]) on the night before. In both evaluations, each participant stood upright as still as possible, in two experimental conditions: fixating the eye on a target and performing saccadic movement toward a target presented in two different locations (0.5 Hz). Each participant performed 3 trials in each condition, lasting 62 s each. Body oscillation was obtained in both anterior–posterior and medial–lateral directions. Results showed that SD participants swayed with a larger magnitude and higher velocity after sleep deprivation in the fixation condition. In the saccadic condition, body sway magnitude and velocity were reduced but were still larger/higher in the SD participants. Sleep deprivation deteriorates the performance of postural control. Saccadic eye movements improve postural control performance even in sleep-deprived participants but are still not sufficient to avoid postural control deterioration due to sleep deprivation.

## Introduction

Sleep conditions have become a recent and relevant problem in modern societies because of the considerable decrease in hours that had been used for resting in the previous decades (Schoenborn and Adams, 2010). Moreover, about one third of adults sleep <6 h per night (Tobaldini et al., 2017), leading to sleep restriction and even sleep deprivation.

Sleep deprivation or restriction affects performance in many of our daily activities. For instance, several studies have shown deleterious effects on postural control performance (Liu et al., 2001; Nakano et al., 2001; Fabbri et al., 2006), with sleep-deprived young adults swaying with larger magnitude (Gribble and Hertel, 2004; Gomez et al., 2008; Patel et al., 2008; Ma et al., 2009; Robillard et al., 2011; Aguiar and Barela, 2014) and higher velocity (Liu et al., 2001; Gribble and Hertel, 2004; Robillard et al., 2011; Aguiar and Barela, 2014) during maintenance of upright stance.

The detrimental effects on postural control performance after sleep deprivation have been attributed to the deterioration of visual-spatial performance (Roge et al., 2002; Kendall et al., 2006; Chee et al., 2010), reduced sensitivity of visual perception (De Gennaro et al., 2000; Fransson et al., 2008), and reduced attention capacity (Lim and Dinges, 2008; Martella et al., 2011; Roca et al., 2012; Vargas et al., 2017). It has also been suggested that sleep deprivation would affect the acquisition of sensory cues and its integration into motor action in maintaining and controlling postural orientation (Fabbri et al., 2006; Gomez et al., 2008; Bougard et al., 2011; Aguiar and Barela, 2014, 2015).

Although postural control functioning is based on sensory cues coming from multiple sources, the role and the use of visual information in postural control functioning and performance have motivated many studies. For instance, a common finding is that the magnitude of oscillation during an upright stance more than doubles when visual cues are absent (Liu et al., 2001; Fabbri et al., 2006; Morad et al., 2007; Ma et al., 2009; Robillard et al., 2011). It has been suggested that the stabilizing effect when fixating a target in the upright stance is due to minimization of the retinal slip of the target projected onto the retina (Paulus et al., 1989) and in doing so, all body sway also would be reduced.

More interesting, however, it is that several studies have shown that postural control performance is even further improved when one performers saccadic eye movements, fixating a target presented in different locations (Rougier and Garin, 2007; Stoffregen et al., 2007; Rey et al., 2008; Legrand et al., 2013; Rodrigues et al., 2013, 2015; Aguiar et al., 2015; Bonnet and Baudry, 2016a,b). Two explanations have been forwarded to account for such postural control improvement due to different visual cues. One explanation suggests that the stabilizing effect of saccadic movements on postural control is due to a possible “efferent copy” of eye movements made available to the central nervous system, which would lead to improvement of postural stabilization (Guerraz and Bronstein, 2008).

The second explanation is that possible reduction of body oscillation would be related to the use of a different goal during the saccadic movement. In this case, to achieve the goal of visual fixation in different targets, one would need to reduce body sway, and thus further postural stabilization would be due to the suprapostural goal of the upright stance task (Stoffregen et al., 1999). In this case, improvement in stability would be achieved, at least in part, to facilitate the performance of the suprapostural task (Oullier et al., 2002).

In both explanations, the postural control system has available extra resources (e.g., sensory cues, attentional efforts, and cognitive enrolment) that lead to performance improvement and more stable upright stance control. Considering that young sleep-deprived adults show less efficient postural control functioning, we question the use of saccade eye movement to improve postural control functioning in sleep-deprived adults. Such questioning is relevant considering that the use of vision in eye-guided movement conditions might be considered an active visual task, involving synergistic relations between the postural and visual systems (Bonnet and Baudry, 2016a,b). Moreover, in such condition the central nervous system needs to cognitively involve both the control of both eye movements and postural sway. Because sleep deprivation or restriction impacts negatively postural control performance that might be due to the acquisition of vision cues (Cheng et al., 2018) and attentional capacity (Caldwell et al., 2003), sleep-deprived adults might prevent the use of additional sensory cues in the eye-guided task or even not be able to synergistically couple eye movements and postural sway, resulting in overall postural control performance improvement. Therefore, the aim of this study was to examine postural control performance of young sleep-deprived adults in fixating and performing horizontal saccades during an upright stance. Our hypothesis was that sleep deprivation would deteriorate postural control performance and also would impair the usage of additional cues from eye-guided movements to improve postural control performance.

## Methods

### Subjects

Twenty-six healthy young adults composed two groups: 13 volunteers constituted the sleep-deprived (SD) group (8 males and 5 females, 24.8 ± 5.8 years) and 13 constituted the control group (CG) (8 males and 5 females, 24.9 ± 5.9 years). Participants were undergraduate students, had normal or corrected-to-normal vision, and reported no diagnosed sleep disturbances or motor commitments. Prior to participation, all volunteers signed a written consent form according to the procedures approved by the Institutional Ethics Committee.

### Procedures

Participants were initially contacted when the experimental procedures were explained. They also were instructed to maintain regular sleep schedules, in the 3 day period before the experimental testing, which were monitored by sleep diaries.

When scheduled, participants from both groups arrived at the laboratory between 8 and 10 a.m., after a normal night of sleep. Each participant was asked to turn in the sleep diaries from the previous days and also to complete the Karolinska Sleepiness Scale (KSS), a scale in which the participant indicated the best level of sleepiness that varies from 1 (extremely alert) to 9 (very sleepy, great effort to keep awake, and fighting sleep). Participants were also asked to complete the Pittsburgh Sleep Quality Index and the Morningness–Eveningness Questionnaire, but these were not used for the purpose of this study. Next, participants underwent the first postural control evaluation (Evaluation 1). After this first testing session, participants from both groups engaged in their regular daily activities throughout the day.

**Figure 1:**
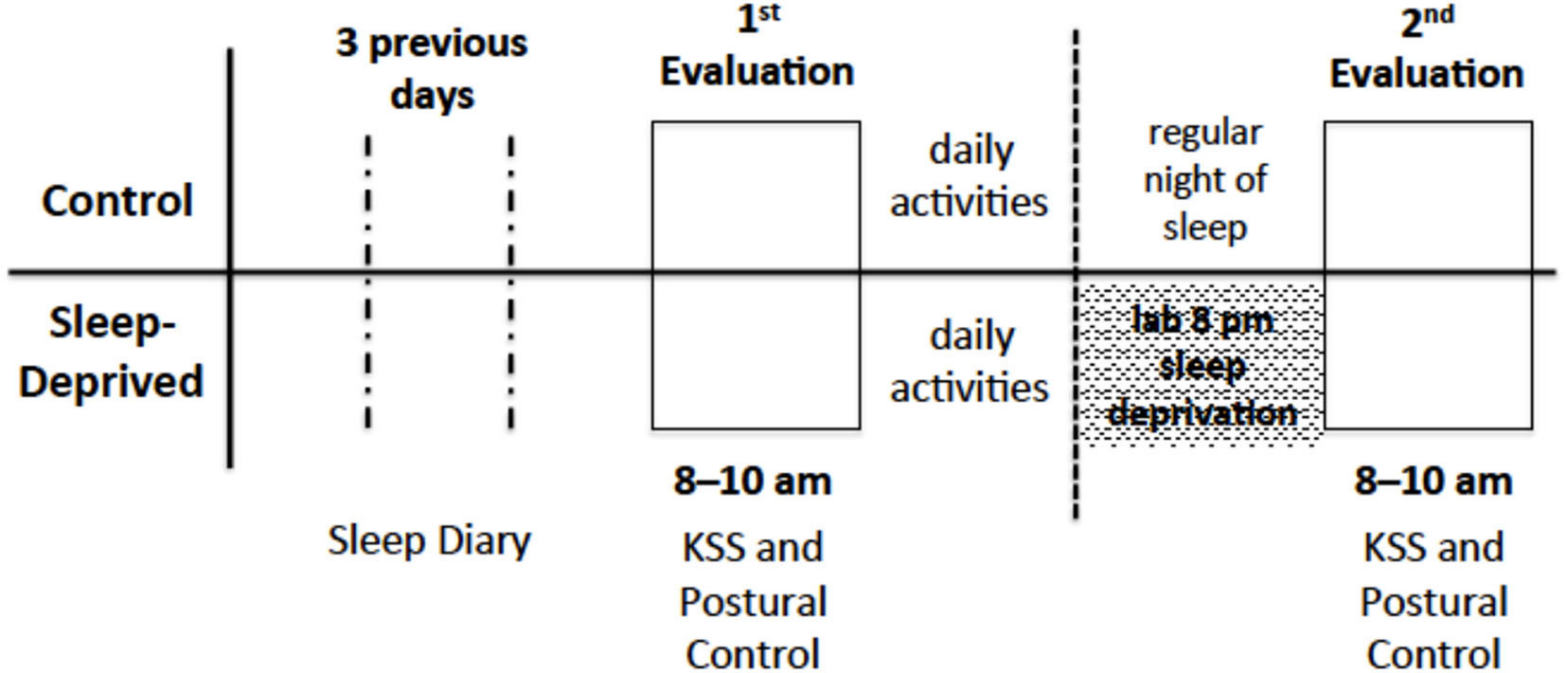
Schematic schedule representation of sleep monitoring and evaluations of participants from both groups.

Participants in the CG were told to sleep as usual and return to the laboratory the next morning, between 8 and 10 a.m., to perform the second postural control evaluation. It was also requested that they would not drink alcoholic beverages and coffee during the night before and prior to coming to the laboratory. Participants in the SD group returned to the laboratory at the end of the day, approximately at 8 pm, and remained awake all night long. Participants were not allowed to drink alcohol and coffee and throughout the night they engaged in activities such as chatting, reading, playing cards and/or games, studying, and watching television. The next morning, between 8 and 10 a.m., participants performed the second postural control evaluation (Evaluation 2). A schematic schedule representation of sleep monitoring and evaluations is shown in Figure 1.

**Figure 2:**
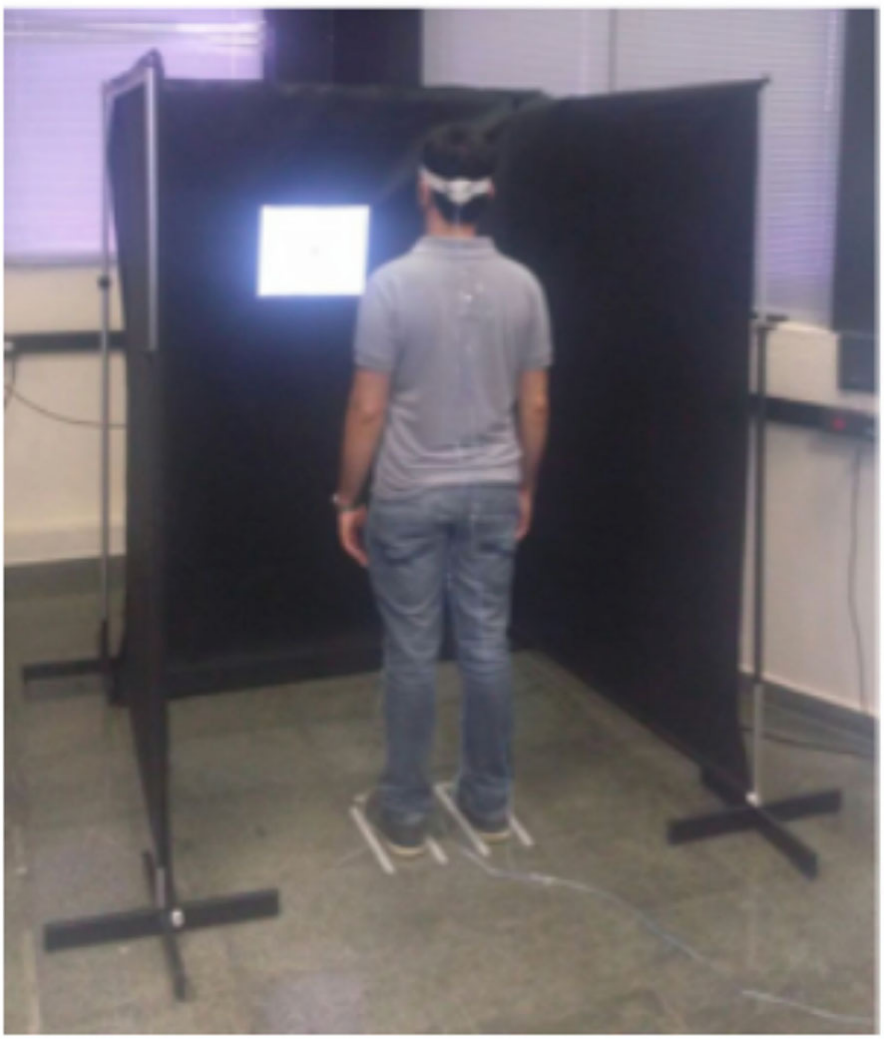
The experimental set up with a participant standing upright, inside the room, with the monitor presented in the frontal wall.

In both evaluations, participants were instructed to stand upright, with feet parallel and apart at waist width, as stable as possible, inside of a room with black curtains (1.5 m long × 1.5 m wide × 1.8 m high), in order to avoid any undesirable visual stimuli from the environment. A screen monitor (LG–Flatron L1753T8) was placed in front of the participant (1 m away) and adjusted at his or her eye level. The experimental setup is depicted in Figure 2. Before maintaining an upright stance, participants wore an eye monitoring system (Eye Tracking Low Cost-dev 1.0) used to automatically track the position of the dominant eye, controlled by specific acquisition software (Pupil Capture, Version 6.3), capturing and displaying online eye position at 30 Hz.

Each participant performed 3 trials, each lasting 62 s, in experimental conditions of fixation and saccades, totaling 6 trials. Participants had a resting interval between trials (about 30 s). In the fixation condition, a target of a 2 cm diameter circle filled in black on a white screen monitor background was presented in the center of the screen for the entire trial. Participants were asked to fixate on it, and the subtended visual angle of the target was ~ 1.15°. In the saccade condition, the same target appeared first on the left side of the monitor, 9.75 cm away from the center, then disappeared and reappeared immediately on the opposite side (i.e., the monitor right side), also 9.75 cm away from the center. The described change in target position occurred constantly in the entire trial, with a frequency of 0.5 Hz, resulting in a total of 31 saccadic eye movements. The total distance between the right and left side targets was 19.5 cm, comprising a visual angle of 11° in the horizontal plane. Participants were asked to follow and fixate the target as quickly as possible, with eye movements and avoiding any head movement. The target appearance, in both conditions, was controlled by specific software (Flash Mx, version 6.0, Macromedia) used in previous studies (Rodrigues et al., 2013, 2015). The first condition, fixation or saccadic, was randomly defined and the following ones were alternated.

Body sway was obtained using an infrared, light-emitting diode (IRED) marker of a motion analysis system (Optotrack Certus, NDI, Bakersfield, CA, USA) placed on each participant's back (around 8th thoracic vertebra level), providing information about position in both anterior–posterior (AP) and medial–lateral (ML) directions. Body sway data were sampled at 100 Hz.

### Data Analysis

Although each trial lasted 62 s, only the intermediate 50 s were considered, with the first and last 6 s periods not considered for analysis. Body sway for both AP and ML directions was filtered using a second-order Butterworth filter with a cut-off frequency of 5 Hz. For each trial, mean sway amplitude and velocity were calculated for both AP and ML directions. Mean sway amplitude was computed as the standard deviation of the positional data throughout the trial, after a first-order polynomial and the mean were subtracted from each data value (detrending). Mean sway velocity was calculated by summing the absolute differences between adjacent positional data of the trial and dividing by the total time of the respective trial. Mean sway amplitude indicated the magnitude of sway variability and mean sway velocity how fast/slow the sway variability occurred.

Eye positioning, in this study, was used only as confirmatory, indicating that participants had accomplished each task requirement, fixation or eye-guided movement. All the procedures were performed using a specific routine written in MATLAB® (MathWorks, Inc., Natick, MA, USA). In addition, the average for each condition was obtained for further analysis.

### Statistical Analysis

Four analyses of variance (ANOVAs), having as factors group (SD and CG), condition (fixation and saccades) and evaluation (first and second), with repeated measures on the last two factors, were conducted. First and second ANOVAs had the mean sway amplitude for the AP and ML directions as the dependent variable, respectively. Third and fourth ANOVAs had the mean sway velocity for the AP and ML directions as the dependent variable, respectively. When any interaction was statistically significant, Tukey HSD *post-hoc* tests were conducted. The significance level was set at 0.05 and all analyses were performed using SPSS software.

## Results

The sleep diaries showed that participants from both groups slept on average similar amounts in both the 3 previous days and in the night before the first evaluation. In contrast, prior to the second evaluation, while participants from the CG had a regular night of sleep, participants from the SD group did not sleep and remained awake. Table 1 depicts the average sleep hours in the previous days and the night before the first evaluation and the time awake prior to the second evaluation for both groups.

**Table 1:** Means (standard deviations) of the sleep hours in the three days before first evaluation, night before first evaluation and hours awaked prior evaluation 2.

	**Day 3 prior evaluation 1**	**Day 2 prior evaluation 1**	**Day 1 prior evaluation 1**	**Night prior evaluation 1**	**Awaked prior evaluation 2**
**Control**	7.21	7.07	6.52	7.06	3.25
	(0.82)	(1.23)	(0.41)	(1.15)	(0.73)
**Sleep-deprived**	7.16	7.09	7.00	7.17	25.82
	(1.33)	(1.43)	(0.87)	(0.80)	(0.86)

Sleep deprivation induced different levels of sleepiness as shown in Table 2, which depicts the Karolinska Scale average values for both groups and evaluations. While the average values indicated alert and relative alert for CG participants in both evaluations, for the SD participants alert and relative alert were indicated in the first evaluation but a state of sleepiness with a great deal of effort to keep awake after sleep restriction and prior to the second evaluation.

**Table 2:** Means (standard deviations) of the Karolinska sleepiness scale values for both control and sleep-deprived participants obtained prior to both evaluations.

	**Evaluation 1**	**Evaluation 1**
**Control**	3.69 (0.95)	3.77 (1.77)
**Sleep-deprived**	3.69 (1.55)	8.38 (0.77)

*Note: Values 3 and 4 indicate alert and fairly alert, respectively, and values 8 and 9 indicate sleepy, some effort to keep alert and very sleepy, great effort to keep alert, fighting sleep, respectively*.

**Figure 3:**
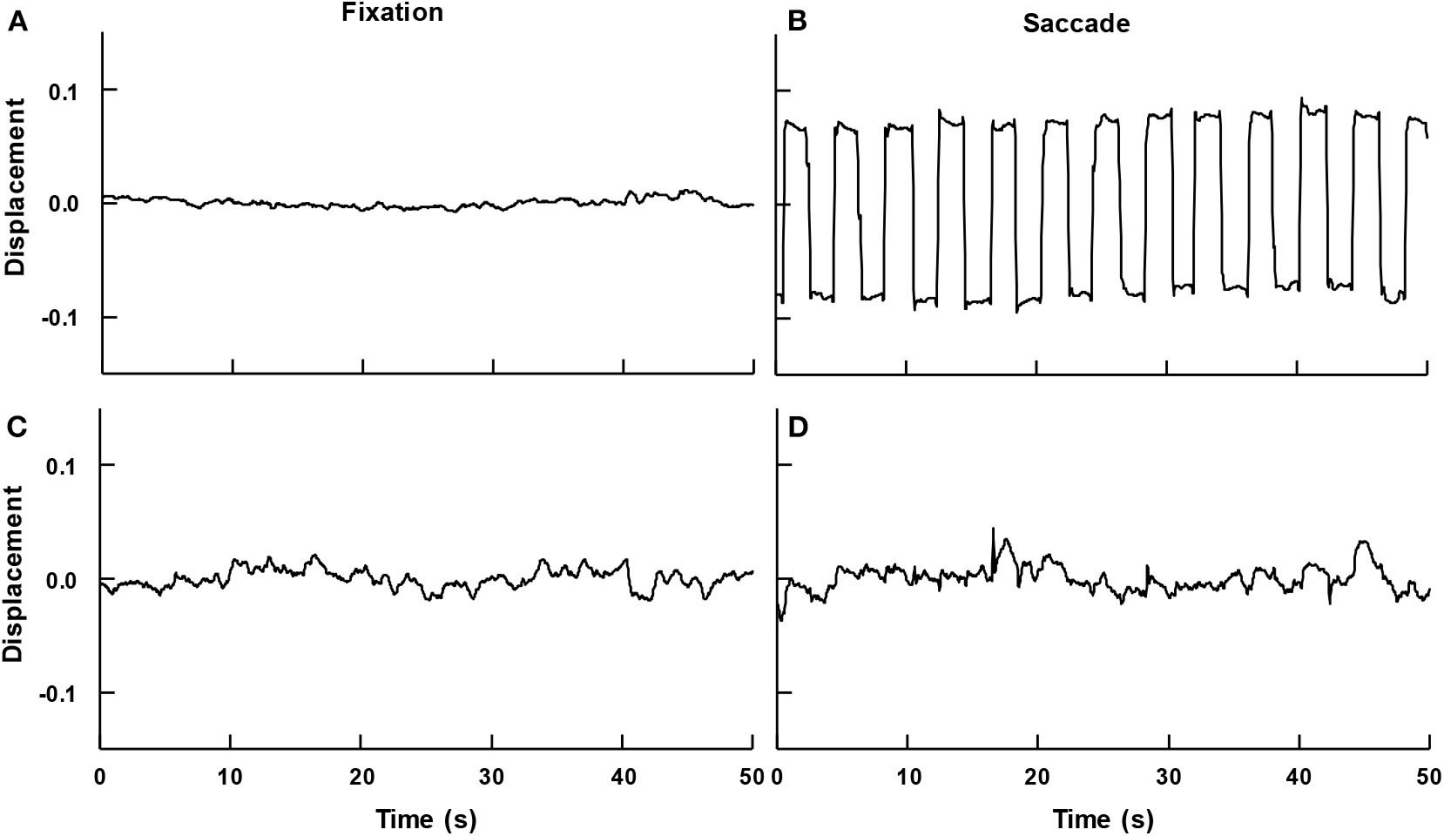
Example time series of a participant's eye movement in the fixation **(A,C)** and in the saccadic **(B,D)** conditions, in the horizontal **(A,B)** and vertical **(C,D)** directions.

Participants were able to maintain an upright stance and to fixate the target displayed in the center to the monitor throughout the trial or to perform saccades and fixate the target as it appeared, disappeared, and again appeared on the other side of the monitor at frequency of 0.5 Hz. Figure 3 depicts an example eye position, vertical and horizontal directions, and time series for both conditions of a participant. As can be seen, in the fixation condition (left panels), horizontal and vertical positions indicate that the eye displayed small displacement as it fixated on the target. On the other hand, in the saccade condition (right panels), the horizontal position indicates eye movements to the left/right direction as the target was also displayed in the left/right side of the monitor. The vertical position also displays small displacement as the target position did not vary up or down.

**Figure 4:**
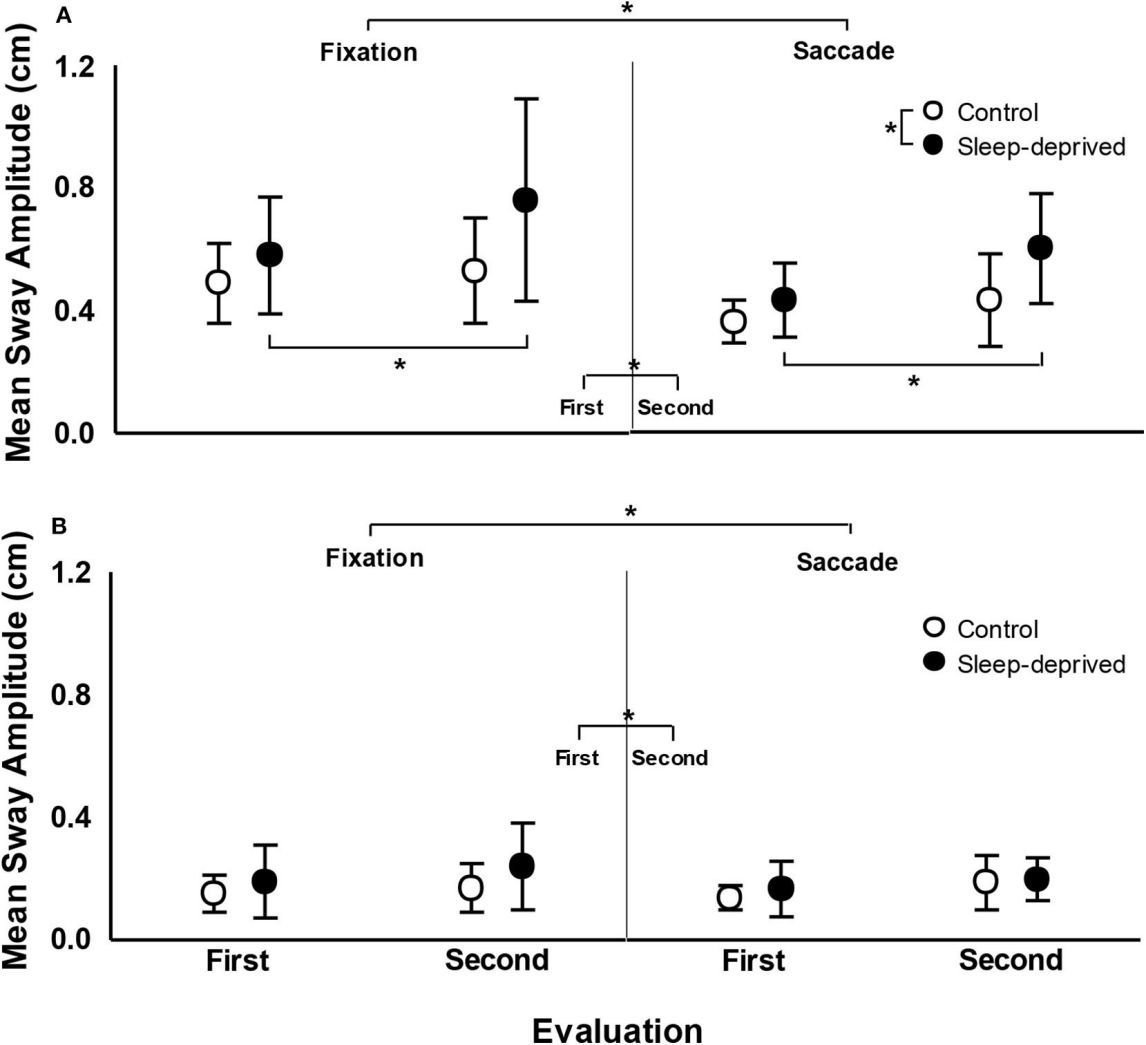
Mean (standard deviation) of mean sway amplitude in the anterior–posterior **(A)** and medial-lateral **(B)** directions in fixation (left) and saccade (right) conditions, in the first and second evaluations, for the control and sleep-deprived groups. Note: *indicates statistical difference.

Figure 4 depicts mean sway amplitude for both SD and CG participants, in both the first and second evaluations, in the fixation and saccade conditions. For the AP direction, ANOVA revealed effect of group, *F*_(1,24)_ = 4.39, *p* < 0.05, $\eta_{p}^{2}$ = 0.155, condition, *F*_(1,24)_ = 25.22, *p* < 0.001, $\eta_{p}^{2}$ = 0.512, evaluation, *F*_(1,24)_ = 17.31, *p* < 0.001, $\eta_{p}^{2}$ = 0.419, and group and evaluation interaction, *F*_(1,24)_ = 4.91, *p* < 0.05, $\eta_{p}^{2}$ = 0.170. Participants with SD swayed with larger magnitude than CG participants; sway magnitude was larger in the fixation than in the saccade condition; in the second evaluation, sway was larger in magnitude than in the first evaluation. Finally, *post-hoc* tests indicated that while CG participants did not differ between first and second evaluations, SD participants swayed with a larger magnitude in the second, after sleep deprivation, compared to the first evaluation. For the ML direction, ANOVA only revealed condition, *F*_(1,24)_ = 5.59, *p* < 0.05, $\eta_{p}^{2}$ = 0.189, and evaluation, *F*_(1,24)_ = 4.73, *p* < 0.05, $\eta_{p}^{2}$ = 0.165, effects. Sway magnitude was larger in fixation than in the saccade condition and was larger in the second than in the first evaluation.

**Figure 5:**
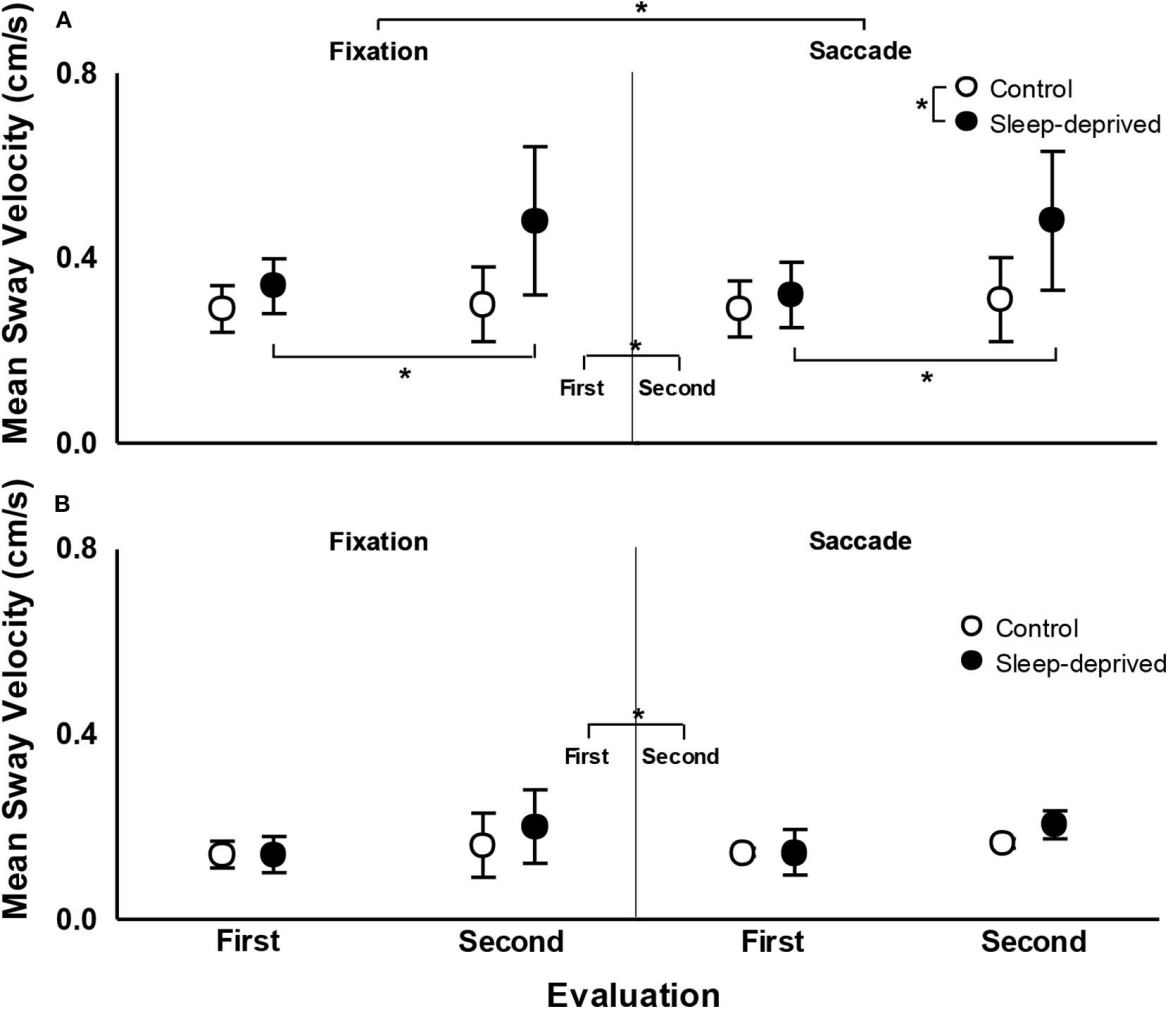
Mean (standard deviation) of mean sway velocity in the anterior–posterior **(A)** and medial-lateral **(B)** directions in fixation (left) and saccade (right) conditions, in the first and second evaluations, for the control and sleep deprived groups. Note: *indicates statistical difference.

Figure 5 depicts mean sway velocity for both SD and CG participants, in both the first and second evaluations, in the fixation and saccade conditions. For the AP direction, ANOVA revealed effect of group, *F*_(1,24)_ = 11.90, *p* < 0.005, $\eta_{p}^{2}$ = 0.332, evaluation, *F*_(1,24)_ = 13.76, *p* < 0.005, $\eta_{p}^{2}$ = 0.364, and group and evaluation interaction, *F*_(1,24)_ = 9.46, *p* < 0.01, $\eta_{p}^{2}$ = 0.283. Participants with SD swayed with higher velocity than CG participants and in the second evaluation sway velocity was higher than in the first evaluation. Finally, *post-hoc* tests indicated that while CG participants did not differ between first and second evaluation, SD participants swayed with higher velocity in the second evaluation, after being sleep deprived, compared to the first evaluation. For the ML direction, ANOVA only revealed effect of evaluation, *F*_(1,24)_ = 6.24, *p* < 0.05, $\eta_{p}^{2}$ = 0.206, and evaluation and condition interaction, *F*_(1,24)_ = 5.14, *p* < 0.05, $\eta_{p}^{2}$ = 0.176. Sway velocity was higher in the second compared to the first evaluation. *Post-hoc* tests did not indicate any pairwise difference for the evaluation and condition interaction.

## Discussion

The goal of this study was to examine upright stance postural control performance of young sleep-deprived adults during continuous fixation and horizontal saccades. Our hypothesis was that sleep deprivation would deteriorate postural control performance and also would impair the usage of additional cues from eye-guided movements to improve postural control performance. Our results corroborate the first part of our hypothesis but refuted the second part, as sleep-deprived participants reduced sway magnitude when using horizontal saccades compared to when only fixating a stationary target. Moreover, sleep deprivation deteriorates postural control performance on a regular basis and despite improving postural control performance, horizontal saccades are not sufficient to overcome the deleterious effects of sleep deprivation, as young sleep-deprived adults still sway with larger magnitude than non-sleep-deprived adults in the AP direction.

Contrary to our hypothesis, results showed that sleep-deprived adults are capable of using saccadic eye movements in order to improve postural control, reducing sway magnitude. Such reduction has been observed for adults not sleep deprived (Stoffregen et al., 2007; Legrand et al., 2013; Rodrigues et al., 2013) and attributed to an attempt to spatially perform the saccades more accurately to the target (Stoffregen et al., 2007) and due to efferent information available to the postural control mechanisms (Guerraz and Bronstein, 2008). Thus, sleep deprivation does not prevent the use of any of the possible mechanisms related to any of these explanations. The improvement in postural control due to eye movement in sleep-deprived adults is relevant considering that it would involve a synergistic relation between the postural and visual systems (Bonnet and Baudry, 2016a), with the central nervous system cognitively involved with both the control of both eye movements and postural sway. In this case, even 24 h of sleep deprivation would not prevent the use of such interaction. The suggestion that sleep deprivation would impact attentional resources (Lim and Dinges, 2008; Martella et al., 2011; Roca et al., 2012; Vargas et al., 2017) requires further understanding because certainly a more complex task such as maintaining an upright stance and producing specific eye movements requires more attention but still provides improvement in postural performance. It might be that postural control and eye movements are not competitive but, conversely, they are congruent (Bonnet and Baudry, 2016a) and the central nervous system still has attentional resources to cognitively couple them.

The impact of sleep deprivation in postural control performance deterioration has been observed in several previous studies (Liu et al., 2001; Nakano et al., 2001; Fabbri et al., 2006) and it was shown in this study as well. Our results clearly showed increased sway magnitude and velocity, in the AP direction, after sleep deprivation when compared to both prior sleep deprivation (within participants) and to not-sleep-deprived participants. Moreover, our results showed that such postural control performance deterioration was observed for both fixation and saccade conditions. Therefore, sleep deprivation deteriorates postural control performance and even the improvement due to eye movement was not sufficient to overcome such a decrease in performance.

Previous studies have generally shown a reduction of approximately one third of body sway magnitude due to saccades as compared to the fixation condition. Mean trunk sway amplitude was reduced in the AP direction between 25 and 30% (Rodrigues et al., 2013) in adults. Additionally, saccadic eye movements consistently reduced postural sway in about the same percentage of young adults in fatigued and unfatigued conditions (Barbieri et al., 2019), in mildly affected people with multiple sclerosis (Santinelli et al., 2019), and even in children with dyslexia (Barela et al., 2020). Interestingly, our results show a similar amount of sway reduction comparing the respective visual conditions, fixation and saccade, to the sleep and sleep-deprived evaluations. In contrast, the sway magnitude observed after sleep deprivation was about the same as that observed in the fixation condition prior to the sleep deprivation. These results indicate that the overall underlying mechanisms related to postural control functioning are in place and working, but after sleep deprivation the magnitude of body sway is already larger and any reduction due to eye-guided movement is not sufficient to overcome the deterioration produced by the absence of sleep.

Recently, it was observed that visual manipulation, inducing postural sway, was maintained after sleep deprivation in young adults but the accuracy with which body sway was produced related to the visual cues and the stability between the visual cues and body sway was clearly affected by the lack of sleep in young adults (Aguiar and Barela, 2014, 2015). The lack of accuracy and stability indicated that individuals with sleep deprivation could couple to the manipulated visual cues but could not uncouple to other sensory cues and, in doing so, their postural control performance was worsened. Results from the present study resemble those in previous ones (Aguiar and Barela, 2014) such that saccadic eye movements were used to improve postural control performance but not to overcome all the deleterious effects of sleep deprivation. Considering that both explanations for using saccadic movements to improve postural control performance are based on the use of additional cues, such enhancement of available sensory cues is not enough to improve performance to the one observed for normal conditions of sleeping. If this is the case, any change and/or impairment in sleep-deprived adults' sensorimotor coupling is still affecting cues coming from the saccadic eye movements. Future studies should aim to carefully examine eye movement characteristics such as velocity, accuracy, and variability of sleep-deprived and control participants. If eye movement characteristics were to be preserved, sleep deprivation would not affect the sensory cues acquisition but instead their usage by the postural control system. Conversely, if eye movement characteristics were to be affected, sleep deprivation would also affect sensory cue acquisition.

In sum, postural control performance of young adults is affected by sleep deprivation. Saccadic eye movements improve postural control stability even after sleep deprivation, but still not to the level observed when there is no sleep deprivation. Therefore, sleep deprivation seems to deteriorate postural control functioning, altering underlying mechanisms that might be overcome by enhanced information furnished by eye movements.

## Data Availability Statement

The datasets generated for this study are available on request to the corresponding author.

## Ethics Statement

All procedures of this study were reviewed and approved by the Cruzeiro do Sul Ethical Committee (CEP-Cruzeiro do Sul #156_2015) and prior to participation, all human volunteers signed a written consent form.

## Author Contributions

IV and LB were responsible to designed the study, obtained and analyzed the data and to organized and wrote the manuscript. SR involved in the discussion and interpretation and revision of the manuscript. JB advised the study development, data analysis and interpretation, and revision of the manuscript. All authors contributed to the article and approved the submitted version.

## Conflict of Interest

The authors declare that the research was conducted in the absence of any commercial or financial relationships that could be construed as a potential conflict of interest.
