# Supplementary figures and images for: Saccadic Eye Movements Attenuate Postural Sway but Less in Sleep-Deprived Young Adults
Source: Front Sports Act Living. 2020 Aug 12;2:97. doi: 10.3389/fspor.2020.00097 (PMC7739768; doi:10.3389/fspor.2020.00097)

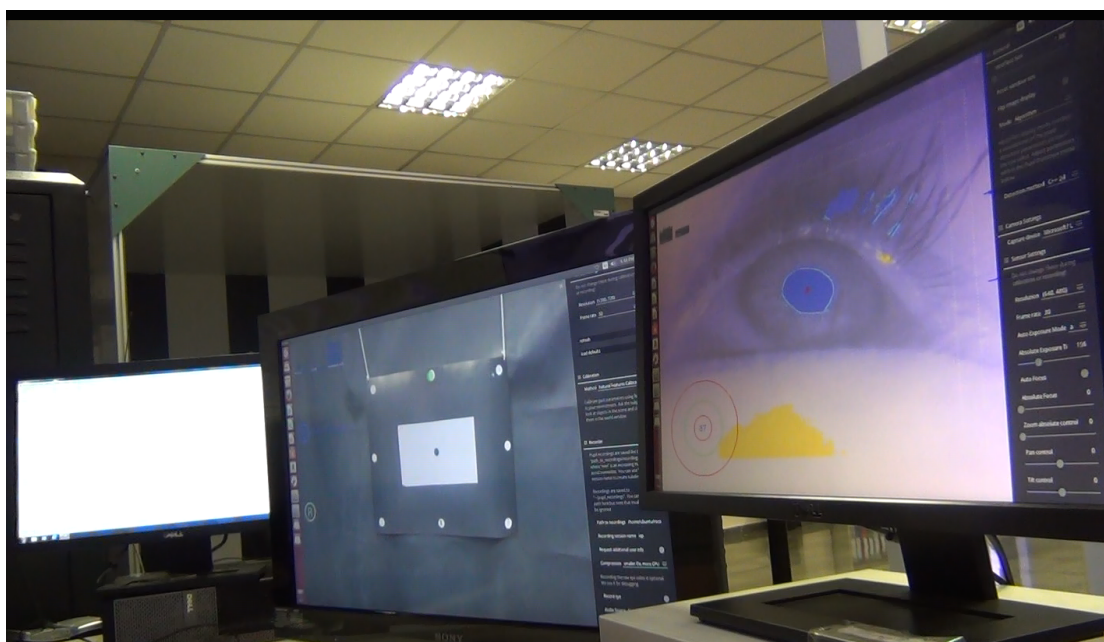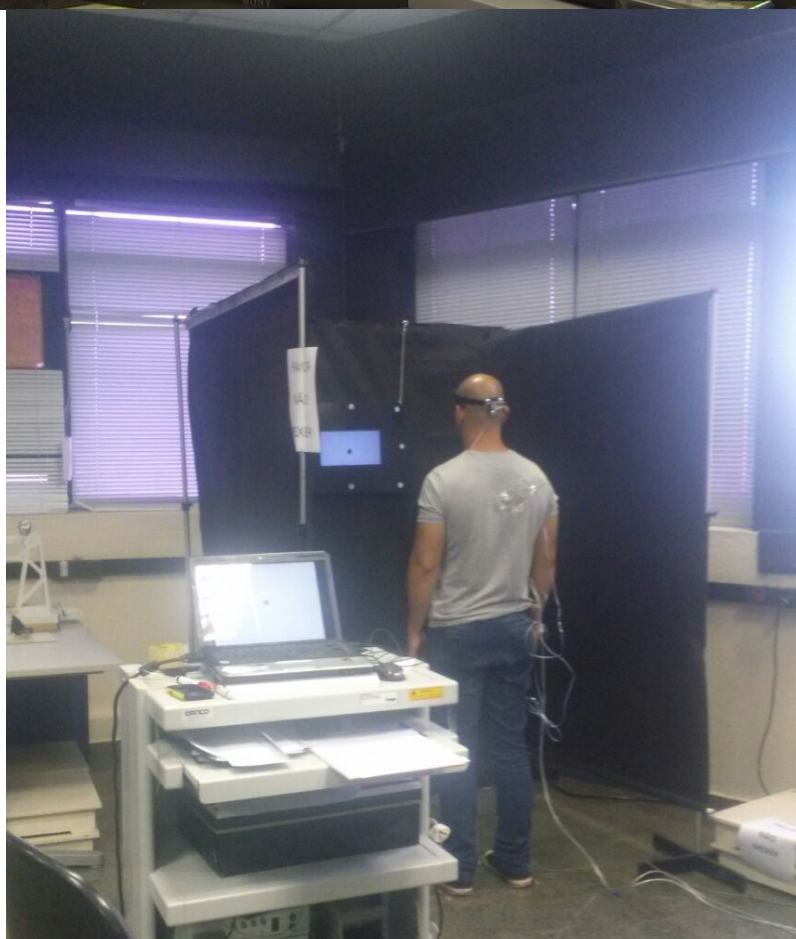

Supplement: Supplementary file 1 [file Data_Sheet_1.PDF]
